# The Surgery of the Kidney

**Published:** 1882-06

**Authors:** 


### THE SURGERY OF THE KIDNEY.

On the 14th of April the Clincal Society finished the long discussion upon abdominal surgery in relation to kidney disease. The chief points raised were :

What cases were suitable for nephrotomy and what for nephrectomy? It was urged by some speakers that the former was a much simpler and safer operation than the latter and ought always to be done first; but others thought that to do so would not always be very easy, because a kidney might be so large as to render it impossible to get it out by the lumbar incision used in nephrotomy; and this gave rise to another point. Is the lumbar operation preferable to Langenbeck's incision along the inner side of the rectus muscle in the abdomen? Mr. Godlee said that the difficulty in getting out the kidney posteriorly was due to adhesions; moreover if the kidney was large the colon may be injured, while deep hæmorrhage cannot be easily controlled posteriorly. In the other method the intestines were hardly seen; and it was recommended by Mr. Clement Lucas that the ureter should be tied before the vessels, then more room would be obtained, as the kidney could be displaced end-ways. He also suggested that in doing the lumbar operation he had found an inverted L-shaped incision gave the best results. Further room might be obtained by pulling up the last rib. Mr. Lucas and Mr. H. Marsh deprecated the use of morphia after operations, until the patient had completely recovered from the anæsthetic, as they thought it added to the risk of collapse. As to age, it appears that under 30 years is best; when the patient is over that, the risk in nephrectomy is increased and then it would probably be better to perform nephrotomy. After the removal of a kidney great care is required to be exercised in regard to the giving of stimulants, so as not to allow collapse to occur, but, on the other hand, not to produce congestion of the remaining kidney. However, uræmia has not been a usual cause of death after the operation. Dr. Southey pointed out that the amount of urea in the urine should be ascertained in each case, and suggested that it would probably be found that those cases would be least successful if the urea was in small amount in proportion to the body weight of the patient.—*N. E. Med. Monthly.*

